# Undifferentiated carcinoma of the liver: a case report with immunohistochemical analysis

**DOI:** 10.1186/s40792-017-0288-0

**Published:** 2017-01-10

**Authors:** Takashi Maeda, Hiroto Kayashima, Daisuke Imai, Kazuki Takeishi, Noboru Harada, Eiji Tsujita, Ayumi Matsuyama, Shinichi Tsutsui, Hiroyuki Matsuda

**Affiliations:** Department of Surgery, Hiroshima Red Cross Hospital and Atomic-bomb Survivors Hospital, 1-9-6 Senda-machi, Naka-ku, Hiroshima 730-8619 Japan

**Keywords:** Undifferentiated carcinoma, Neuroendocrine carcinoma, Liver cancer

## Abstract

**Background:**

Undifferentiated carcinoma (UC) of the liver is extremely rare, and its clinicopathological characteristics have not been fully elucidated.

**Case presentation:**

The present study reports the case of a 56-year-old male with UC of the liver. At 16 days post-admission, the patient suddenly succumbed due to intra-abdominal bleeding resulting from a ruptured liver. Macroscopic examination revealed that the hepatic parenchyma was almost completely replaced by innumerable minute nodules. Microscopically, the tumor demonstrated a diffuse proliferation of anaplastic cells that were positive for epithelial membrane antigen and neuron-specific enolase, suggesting neuroendocrine differentiation. Grimelius and Fontana-Masson staining were negative. Neuroendocrine markers were also negative, including chromogranin A, synaptophysin, and S-100 protein. Cytokeratin markers and mesenchymal markers were all negative. Tumor markers, such as *α*-fetoprotein and carcinoembryonic antigen, were also negative.

**Conclusions:**

Although larger studies involving more patients are required to establish a therapeutic strategy, appropriate chemotherapy following an early diagnosis may be important to UC of the liver because the tumor behaves in an aggressive way.

## Background

Undifferentiated carcinoma (UC) of the liver is an extremely rare malignant neoplasm [1, 2]. The clinicopathological characteristics and clinical course of UC have not been fully clarified, and a therapeutic strategy for unresectable cases has not been established. The present study reports the case of a 56-year-old male with UC of the liver, together with the results of immunohistochemical analysis.

## Case presentation

**Fig. 1:**
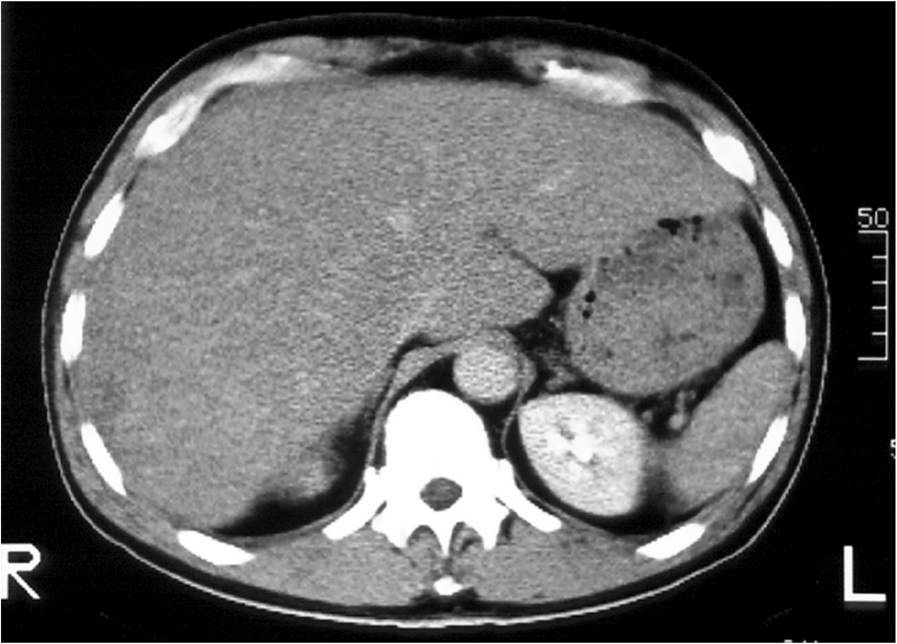
Computed tomography. The liver was remarkably swollen and the parenchyma was diffusely rough

A 56-year-old male with chronic hepatitis was admitted due to hematemesis and general fatigue. The patient had undergone a distal gastrectomy with Billroth I reconstruction for duodenal ulcer 16 years previously. He had no history of blood transfusion and alcohol abuse. Upon physical examination, anemia, jaundice, and hepatomegaly were noted. The laboratory findings upon admission were as follows: hemoglobin, 10.8 g/dl; hematocrit, 32.4%; platelet count, 211 × 10^3^/μl; white blood cell count, 13.6 × 10^3^/μl; serum total protein, 5.7 g/dl; serum albumin, 3.2 g/dl; *γ*-globrin, 14.1%; total bilirubin, 2.7 mg/dl; serum aspartate aminotransferase, 150 IU/l; serum alanine aminotransferase, 129 IU/l; serum glutamyltransferase (γ-GT), 474 IU/l; serum alkaline phosphatase (ALP), 833 IU/l; and NH_3_, 84 μg/dl. Viral markers for hepatitis were negative, including hepatitis B surface antigen and hepatitis C viral antibody. The α-fetoprotein (AFP) was 3.8 ng/ml (normal range <20 ng/ml) and PIVKA-II was normal (<0.06). A chest X-ray revealed neither a mass lesion nor pleural effusion. Gastrointestinal endoscopy revealed a hemorrhagic ulcer at the anastomosis. Computed tomography revealed marked swelling of the liver and a hepatic parenchyma that was diffusely rough (Fig. 1). It was difficult to distinguish tumorous area from non-tumorous area because tumor margin was obscurely demonstrated. A small amount of ascites was found. Abdominal ultrasonography revealed a 6 × 6 cm isoechoic vague mass at the postero-inferior segment of the liver. However, it was difficult to distinguish tumorous area from non-tumorous area by also ultrasonography, and the tumor seemed to spread through the whole liver. No abnormalities were demonstrated in the other organs, including the gallbladder and pancreas. Therefore, this was suspected to be a case of a diffuse type of hepatocellular carcinoma, although an abdominal angiography could not be performed due to the poor general status of the patient. A fine-needle aspiration biopsy specimen of the liver showed a diffuse proliferation of anaplastic cells with hyperchromatic nuclei and scant cytoplasm, suggesting undifferentiated carcinoma of the liver.

**Fig. 2:**
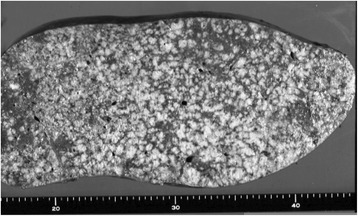
Macroscopic finding. The hepatic parenchyma was almost completely replaced by innumerable minute nodules

**Fig. 3:**
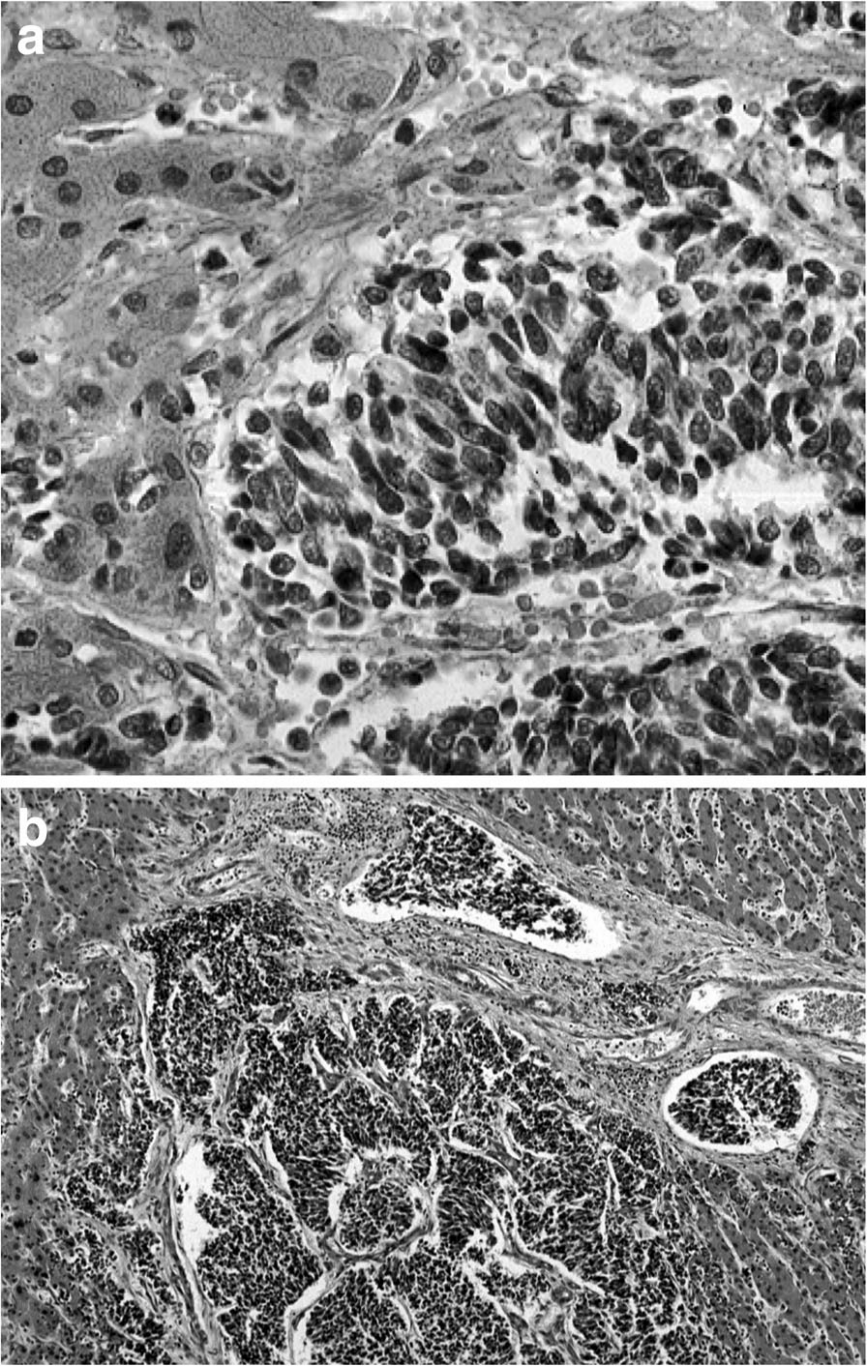
Microscopic finding. **a** A diffuse proliferation of anaplastic cells with hyperchromatic nuclei and scant cytoplasm, with frequent mitotic figures. **b** Conspicuous portal venous invasion of the tumor cells

Following admission, the patient developed severe hematemesis and the hemoglobin level decreased to 7.0 g/dl. Jaundice rapidly progressed, and the ascites increased. At 16 days post-admission, the patient suddenly succumbed due to intra-abdominal bleeding from a ruptured liver. On autopsy, the weight of the liver was 7.5 kg. The macroscopic examination revealed that the hepatic parenchyma had almost totally been replaced by innumerable minute nodules (Fig. 2). Microscopically, a diffuse proliferation of anaplastic cells with hyperchromatic nuclei and scant cytoplasm was observed (Fig. 3), indicating UC of the liver. In addition, mitotic figures were frequently present.

**Fig. 4:**
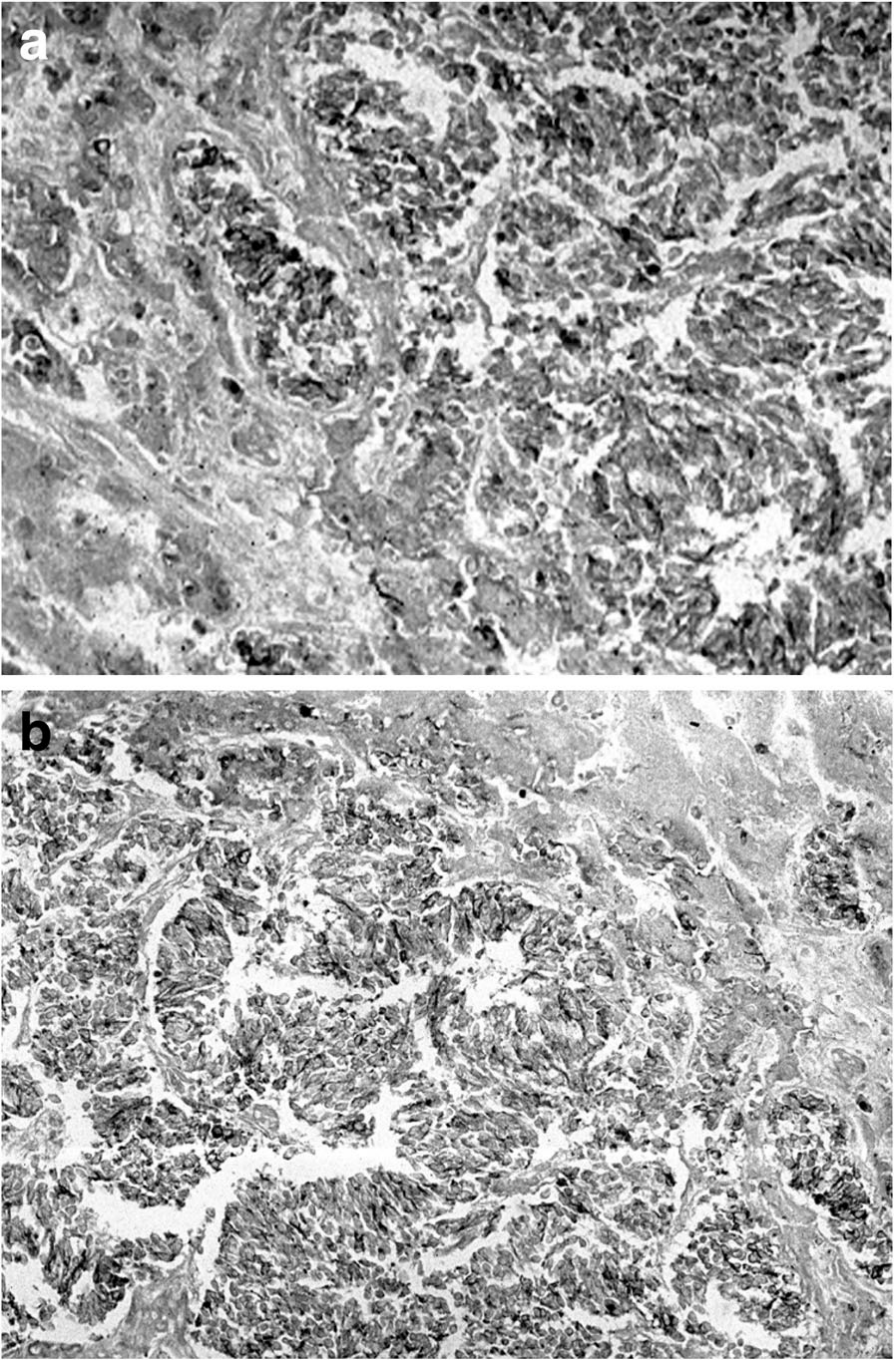
Immunohistochemical staining. **a** Neuron-specific enolase was focally positive. **b** Epithelial membrane antigen was diffusely positive

Immunohistochemical studies were performed using the avidin-biotin-peroxidase complex technique. Immunopositivity was described as follows: (i) negative (−) when <10% of the tumor cells were positive, (ii) focally positive (1+) when 10–50% of the tumor cells were positive, and (iii) diffusely positive (2+) when >50% of the tumor cells were positive. Tumor markers were negative, including AFP, carcinoembryonic antigen, carbohydrate antigen 19-9, and squamous cell carcinoma antigen, whereas neuron-specific enolase (NSE) was focally positive (Fig. 4a). Epithelial membrane antigen was diffusely positive (Fig. 4b), whereas cytokeratin (CK) markers (e.g., CK7, CK8, CK18, CK19, CK20, AE1/AE3, CAM 5.2, CK 902, and CK 903) were all negative. Grimelius and Fontana-Masson staining were negative. Neuroendocrine markers were also negative, including chromogranin A, synaptophysin, and S-100 protein. Additional mesenchymal markers were negative as well, such as vimentin, desmin, *α*-smooth muscle actin, HHF-35, KP-1, and CD34.

### Discussion

UC of the liver is an extremely rare malignant neoplasm, and its clinicopathological characteristics have not been fully elucidated. Only a few case studies have been published [1, 2] (Table 1). Nakasuka et al. [1] reported the case of a patient with UC of the liver wherein a biopsy revealed a UC pattern with neuroendocrine features. Immunohistochemical analysis revealed immunoreactivity to the synaptophysin endocrine marker. Additionally, the serum NSE level was high at the time of diagnosis, although NSE stain positivity was not specified. Therefore, the patient was diagnosed with UC of the liver with neuroendocrine features. Several immunohistochemical studies in primary liver cancers with neuroendocrine differentiation have been reported [3–7]. The World Health Organization tumor classification states the synaptophysin and chromogranin A neuroendocrine markers are usually expressed in high-grade neuroendocrine carcinoma [8]. Furthermore, abnormal serum NSE and synaptophysin levels are considered to be additional evidence suggesting neuroendocrine differentiation.

**Table 1:** Summary of reported cases of undifferentiated hepatocellular carcinoma of the liver

Case	Age	Sex	Tumor markers	US/CT	Immunohistochemistry	Chemotherapy	Prognosis
1. [1]	54	M	WNL	Multiple <3 cm	Synaptophysin	Etoposide + cisplatin	Alive (8 m)
2. [2]	57	M	WNL	Solitary = 10 cm	CAM5.2, HepPar1, CK19, vimentin	Unknown	Unknown
3. [Present case]	56	M	WNL	Diffuse	NSE, EMA	ND	Died (16 days)

Extrapulmonary small cell carcinomas are extremely rare and can originate in a variety of sites, including the liver, colon, stomach, esophagus, cervix, gallbladder, and skin [9–14]. Zanconati et al. [9] reported three cases of unusual small cell primary carcinoma of the liver. All three tumors developed in non-cirrhotic livers and demonstrated rapid clinical evolution, with mortality ensuing between 1 and 5 months post-diagnosis. These tumors were comprised of broad nests of small epithelial cells positive for low-molecular weight keratins and AFP.

In the present case, the tumors were growing in a solid nest comprising small cells with hyperchromatic nuclei and scant cytoplasm, which indicated small cell carcinoma. By contrast, immunohistochemical analysis revealed positive staining for NSE, although it was negative for chromogranin A and synaptophysin, therefore suggesting neuroendocrine differentiation. It is difficult to determine which classification is appropriate for this type of tumor. The tumor is referred to as UC with neuroendocrine features, neuroendocrine carcinoma, or small cell carcinoma of the liver. Thus, a novel entity of the tumor should be established for the accumulation of such cases in the future.

UC grows invasively, features a high incidence of metastasis, and follows a rapid clinical course [2]. However, no therapeutic strategies for unresectable tumors have been established. Moertel et al. [15] reported the chemotherapeutic results for metastatic UC with endocrine features and concluded that this type of tumor responded strongly to etoposide and cisplatin regimen chemotherapy. Similarly, Nakasuka et al. [1] reported a case of UC of the liver with neuroendocrine features treated with the same regimen that resulted in a positive clinical course. Other studies reported poorly differentiated/rapidly progressing neuroendocrine tumors [16] and poorly differentiated neuroendocrine carcinoma of the hepatobiliary tract and pancreas [17], which were treated with a combination of cisplatin and etoposide.

## Conclusions

We herein reported the case of a 56-year-old male with UC of the liver. This tumor demonstrated a diffuse proliferation of anaplastic cells, which were positive for NSE suggesting neuroendocrine differentiation, and showed an aggressive behavior. Therefore, appropriate chemotherapy following an early diagnosis may be important to UC of the liver. In the future, larger studies on UC of the liver should be conducted to analyze an appropriate chemotherapeutic strategy for this rare disease.
